# Comorbidity in Adult Patients Hospitalized with Type 2 Diabetes in Northeast China: An Analysis of Hospital Discharge Data from 2002 to 2013

**DOI:** 10.1155/2016/1671965

**Published:** 2016-10-25

**Authors:** Hui Chen, Yaoyun Zhang, Di Wu, Chunxiu Gong, Qing Pan, Xiao Dong, Yonghui Wu, Kuan Zhang, Shiping Wang, Jianbo Lei, Hua Xu

**Affiliations:** ^1^School of Biomedical Engineering, Capital Medical University, Beijing, China; ^2^Beijing Key Laboratory of Fundamental Research on Biomechanics in Clinical Application, Capital Medical University, Beijing, China; ^3^School of Biomedical Informatics, The University of Texas Health Science Center at Houston, Houston, TX, USA; ^4^Department of Endocrinology, Genetics, and Metabolism, Beijing Children's Hospital, Capital Medical University, Beijing, China; ^5^Health Information Center, Dalian, Liaoning, China; ^6^Health Science Center, Peking University, Beijing, China; ^7^School of Medical Informatics and Engineering, Southwest Medical University, Luzhou, Sichuan, China

## Abstract

This study aims to evaluate the comorbidity burden and patterns among adult patients hospitalized with a diagnosis of type 2 diabetes mellitus (T2DM) in Northeast China using hospital discharge data derived from the electronic medical record database between 2002 and 2013. 12.8% of 4,400,892 inpatients aged ≥18 had a diagnosis of T2DM. Sex differences in prevalence varied among those aged <50, 50–59, and ≥60. Twenty-seven diseases were determined as major comorbidities of T2DM. Essential hypertension was the most common comorbidity of T2DM (absolute cooccurrence risk, 58.4%), while T2DM was also the most popular comorbidity of essential hypertension. Peripheral and visceral atherosclerosis showed the strongest association (relative cooccurrence risk, RCoR 4.206). For five leading comorbidities among patients aged ≥40, male patients had a stronger association with disorders of lipid metabolism than female patients (RCoR 2.779 versus 2.099), and female patients had a stronger association with chronic renal failure than male patients (RCoR 2.461 versus 2.155). Leading comorbidities, except chronic renal failure, had declining associations with T2DM with increased age. Collectively, hospital discharge data can be used to estimate disease prevalence and identify comorbidities. The findings provided comprehensive information on comorbidity patterns, helping policy makers and programs in public health domains to estimate and evaluate the epidemic of chronic diseases.

## 1. Introduction

The prevalence of diabetes is increasing worldwide [1]. Clinical cross-sectional study and cohort study revealed that patients with type 2 diabetes mellitus (T2DM) are at increased risk of cardiovascular and cerebrovascular diseases and associated clinical complications, leading to diabetes being a major cause of premature illness and death. It is predicted that, by 2030, T2DM will be the seventh leading cause of death in the world [2]. Therefore, precise and clear understanding of the epidemiology of diseases that coexist with diabetes, especially chronic illnesses, is important for setting treatment goals.

While patients with T2DM are at increased risk of comorbidity, few data sources are available for evaluating the comorbidity burden and patterns among patients with T2DM. Many population-based surveys and clinical studies have attempted to determine how T2DM affects the risk of cardiovascular and cerebrovascular diseases and associated complications [3–5], focusing on specific disorders related to T2DM, such as cardiovascular autonomic neuropathy [6, 7], pulmonary tuberculosis [8], and chronic kidney disease [9], and/or on specific populations with T2DM, such as patients with dementia [5], the elderly [10], and people with depression [11]. Clinical studies may have inconsistent findings because of relatively small sample sizes and variations in sample characteristics and settings [12], whereas survey data usually focus on specific disorders and sometimes include inadequate information on diagnoses and treatment. Therefore, there is a need for comprehensive information from large long-term datasets to improve understanding of the prevalence of T2DM-related comorbidities, along with subgroup analysis.

With the emergence of the big data era, national or regional adoption of electronic medical records (EMR) systems has improved the efficiency and quality of healthcare delivery and allowed the opportunity to use real-world patient information for clinical data mining. EMR data have become a priority for research on disease relationships, such as assessing comorbidities of substance use [13, 14], studying temporal relationships between T2DM and cancer [15], analyzing disease networks [16], and modeling to predict disease severity [17] and to identify patients [18, 19]. Hospital discharge data, as a kind of administrative data derived from EMR, allow investigators access to a broad range of illness, whose discharge diagnosis codes are assigned by trained doctors following standard guideline. Therefore, hospital discharge data are becoming one of the available data sources for assessing hospital prevalence and comorbidity for a specific disease [20–22]. However, to our best knowledge, none of these studies has focused on analyzing the trend in both the prevalence and comorbidity patterns with respect to T2DM.

China has the largest number of individuals with diabetes in the world. In 2014, the prevalence of T2DM was estimated at 9.32% among the adult Chinese population aged 18–79 years, representing an estimated 96.3 million people [23]. China is estimated to have approximately 143 million T2DM patients by 2035 [23]. However, most current epidemic information about T2DM in China was collected through surveys [24–27]. Few studies [28, 29] have utilized real-world data from a single hospital to assess T2DM prevalence and/or comorbidity in China. On the contrary, the Chinese government has invested huge amounts of funding to deploy EMR systems at hospitals across the nation in the past decade. EMRs are expected to be deployed and implemented nationwide in all public hospitals at county level and above by 2017 [30]. The rapid implementation of EMRs in China has accumulated huge amounts of clinical data, which are suitable for answering questions such as T2DM prevalence and comorbidity.

In this study, we used a large administrative database (involving 4,123,405 patients), which includes hospital discharge information derived from EMRs of all hospitals in a large city in Northeast China during 2002 through 2013, to estimate the risk of T2DM-related comorbidities, as well as their trends along the timeline. We believe this is the first study that utilizes large EMR-derived data to assess T2DM status in China, especially in Northeast China. We hope this study also serves as a new model for better understanding diseases using real-world data.

## 2. Materials and Methods

### 2.1. Data Source and Study Population

Hospital discharge data were derived from EMR databases of all hospitals in Dalian, China, from January 2002 to December 2013. Dalian is the second largest city in Northeast China, with 6.9 million permanent residents in 2013. The dataset contained more than 6 million records, including demographic information (sex and date of birth), date of admission, date of discharge, one primary discharge diagnosis, and up to 5 secondary discharge diagnoses. Data for patients aged ≥18 years were deidentified and included in this study. The use of these data in an anonymous manner was authorized by the Information Center, Health and Family Planning Commission of Dalian Municipality.

All diagnoses were identified with International Classification of Diseases, Tenth Revision (ICD-10) codes [31]. These diagnostic codes were then recoded into one of 259 categorization codes defined by Clinical Classifications Software (CCS) for ICD-10-CM [32], which is a diagnosis categorization scheme based on ICD-10 codes. CCS codes are diagnosis categories with more clinical meanings, which can sometimes be more useful for presenting descriptive statistics than individual ICD-10 codes for relatively specific conditions. They are now widely used in many study scenarios for identifying comorbidities and outcomes [33–35], predicting mortality and risk [36, 37], and estimating hospital utilization and costs [38].

### 2.2. Statistical Analysis

All samples were stratified by age, sex, and calendar year. Age in years was categorized into following groups: 18–29, 30–39, 40–49, 50–59, 60–69, 70–79, and ≥80.

In each individual medical record with two and above diagnoses, all possible disease pairs among these diagnoses were extracted. For specific diseases *X* and *Y*, a two-by-two table was constructed as seen in Table 1 where *a* and *b* are numbers of records having disease *X* with and without *Y*, respectively, and *c* and *d* are numbers of records not having disease *X* with and without *Y*, respectively. The absolute cooccurrence risk (ACoR) of disease *Y* in condition of *X* was calculated as *a*/(*a* + *b*), and the relative cooccurrence risk (RCoR) of disease *Y* was calculated as the ACoR of disease *Y* with *X* divided by the ACoR of disease *Y* without *X*; that is, RCoR = (*a*/(*a* + *b*))/(*c*/(*c* + *d*)).

Major T2DM-related comorbidities were defined as disease *Y* with both ACoR > 1% and RCoR > 1 in the condition of T2DM. Both ACoR and RCoR were also calculated by sex, age, and calendar year. The changing trends of RCoRs from 2002 to 2013 were described and analyzed. Mann–Whitney *U* tests were used to compare RCoR differences between men and women, and Kruskal–Wallis tests were used to compare RCoR differences among age groups. Major comorbidities were grouped into several categories by using a hierarchical cluster analysis (Ward's minimum-variance method with Euclidean distance measure) conducted on their yearly ranks of RCoRs. To reduce the probability of type I error, differences were considered significant at *P* < 0.001.

All statistical analyses were performed using open source package R 3.2.3 (the R Project for Statistical Computing, https://www.r-project.org/).

## 3. Results and Discussion

### 3.1. Overview of the Study Population with T2DM

Overall, there were 4,400,883 patients (2,072,348 men and 2,328,535 women) aged ≥18 years discharged between 2002 and 2013, of whom 12.8% had a diagnosis of T2DM (12.9% in women and 12.7% in men).

**Figure 1:**
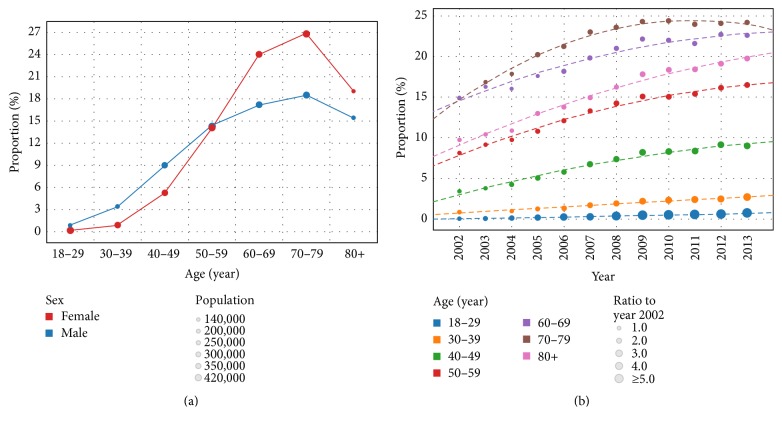
Trends in proportion of hospitalized patients with type 2 diabetes mellitus among all discharged inpatients. (a) The color and size of circles show details on sex and population, respectively. (b) The size of the circle representing the proportion in year 2002 was set at 1, and the sizes of circles representing proportions of other years were defined as the proportion ratios compared with those in year 2002.

Figure 1(a) shows the proportions of hospitalized T2DM patients among the study population stratified by sex and age. Proportion of men and women with T2DM varied for different age groups; that is, proportions of men with T2DM aged <50, 50–59, and ≥60 years were lower than, similar to, and higher than those of age-paired women, respectively. Proportion of hospitalized T2DM patients increased with age up to 80 years, followed by a small reduction in ≥80 years' age group.

From 2002 to 2013, proportion of hospitalized T2DM patients increased from 6.5% to 15.7% overall, representing a 2.4-fold increase (*P* < 0.001). When stratified by age, the proportions increased 7.7-fold, 3.0-fold, 2.6-fold, 1.5-fold, 1.6-fold, 1.6-fold, and 2.0-fold for patients aged 18–29, 30–39, 40–49, 50–59, 60–69, 70–79, and ≥80, respectively. Proportion of hospitalized T2DM patients showed the fastest increment among people aged 18–29 years (Figure 1(b)).

### 3.2. Overall Comorbid Disorders Associated with T2DM

**Figure 2:**
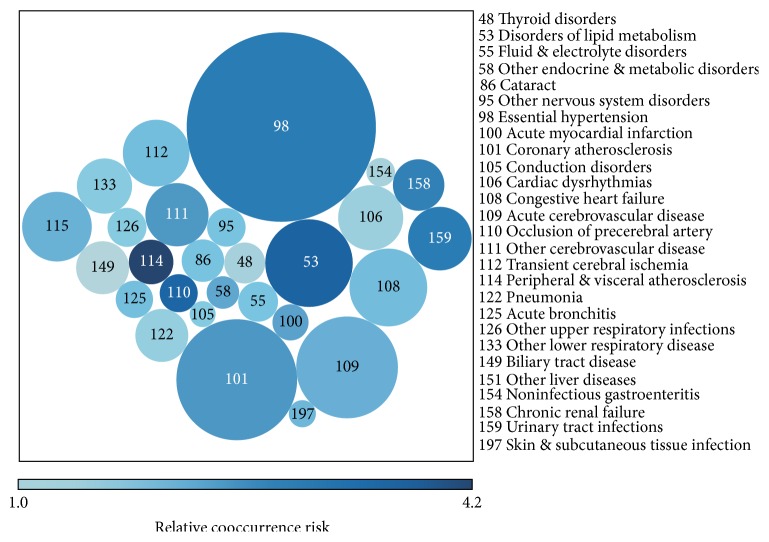
Twenty-seven major comorbidities with absolute cooccurrence risk (ACoR) > 1% and relative cooccurrence risk (RCoR) > 1. The size and color of circles show the ACoR and RCoR, respectively. Larger circles represent higher ACoRs and darker circles represent higher RCoRs.

Twenty-seven diseases were determined as overall major comorbidities, having both ACoRs > 1% and RCoRs > 1 (Figure 2 and Table S1 in Supplementary Material available online at http://dx.doi.org/10.1155/2016/1671965). Essential hypertension (EH), coronary atherosclerosis and other heart diseases (CHD), and acute cerebrovascular disease (ACVD) were the top three comorbidities with the largest ACoRs (58.4%, 23.9%, and 16.9%, resp.), while peripheral and visceral atherosclerosis (PVA), disorders of lipid metabolism (DLM), and occlusion or stenosis of precerebral arteries were the top three comorbidities with the highest RCoRs (4.206, 3.477, and 3.409, resp.). Nonspecific comorbid disorder showed the largest ACoR and RCoR at the same time.

**Figure 3:**
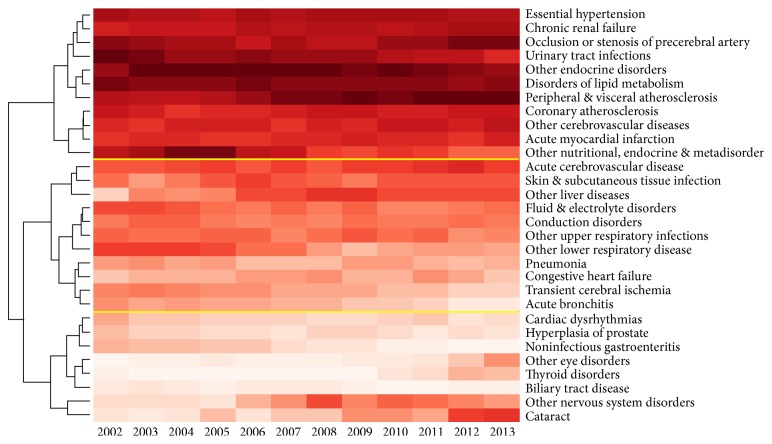
Results of ranking and cluster analysis on ranks of 27 overall major comorbidities. Comorbidities with a higher relative cooccurrence (RCoR) risk were ranked to a smaller number in a darker color and those with a lower RCoR were ranked to a larger number in a lighter color.

Then, 27 overall major comorbidities were ranked from 1 with the largest RCoR to 27 with the smallest RCoR for each year during 2002–2013.  Figure 3 shows the results of ranking and cluster analysis conducted on ranks. Twenty-seven overall major comorbidities could be clustered into three categories with high (represented by DLM and PVA), medium (represented by skin and subcutaneous tissue infections and ACVD), and low (represented by cardiac dysrhythmias and noninfectious gastroenteritis) RCoRs, respectively. During the 2002–2013 period, comorbidities with the top RCoRs varied for each year, resulting in three patterns of comorbidity ranks for these 12 years, that is, years 2002–2005 with top three largest RCoRs for other nutritional, endocrine, and metabolic disorders, urinary tract infection (UTI), and DLM; years 2006–2011 with the top three largest RCoRs for UTI, DLM, and PVA; and years 2012-2013 with the top three largest RCoRs for DLM, PVA, and occlusion or stenosis of precerebral arteries.

Although the associations between 27 overall major comorbidities and T2DM changed during the study period, we noted that 10 diseases, including two endocrine and metabolic disorders (DLM and other nutritional, endocrine, and metabolic disorders (NEMD)), six circulatory system disorders (e.g., EH, CHD, and PVA), and two genitourinary system disorders (CRF and UTI), showed a strong association with T2DM along time. Among these diseases, other NEMD and UTI showed a decreasing association with T2DM over time, while PVA and occlusion or stenosis of precerebral arteries showed an increasing association, reflecting the impacts of lifestyle and dietary habits, as well as a growing awareness of the comorbidities among T2DM patients. Moreover, diseases with weak associations with T2DM, such as cataract and other nervous system disorders, also showed an increasing association with T2DM. Once developed, these microvascular complications of diabetes are mostly irreversible and they should therefore be a focus of attention in awareness and prevention programs, alongside hypertension, which frequently contributes to the development of microvascular complications.

### 3.3. Sex and Age Differences of Associations between T2DM and Related Comorbidities

When taking patient sex or age into consideration, major comorbidities varied for the particular populations. Twenty-two out of 27 overall major comorbidities, such as EH, DLM, and chronic renal failure (CRF), remained the major comorbidities for both male and female patients, whereas biliary tract disease and noninfectious gastroenteritis for male patients and thyroid disorders plus other two diseases for female patients could no longer be considered as major comorbidities because of their RCoRs not reaching >1 or ACoRs not reaching >1% statistically. However, some diseases, for example, hyperplasia of prostate and chronic obstructive pulmonary disease, became major comorbidities for male and female patients, respectively (Table S2).

Regarding patient age, only 15, 19, 24, 21, 19, 17, and 18 diseases out of 27 overall major comorbidities remained to be major comorbidities for patients aged 18–29, 30–39, 40–49, 50–59, 60–69, 70–79, and ≥80 years, respectively (Table S3). Some diseases could be considered as major comorbidities for specific age groups, for example, gastritis and duodenitis for patients aged 18–39 years, tuberculosis and hepatitis for patients aged 30–49 years, and senility and organic mental disorders for patients aged ≥80 years.

**Figure 4:**
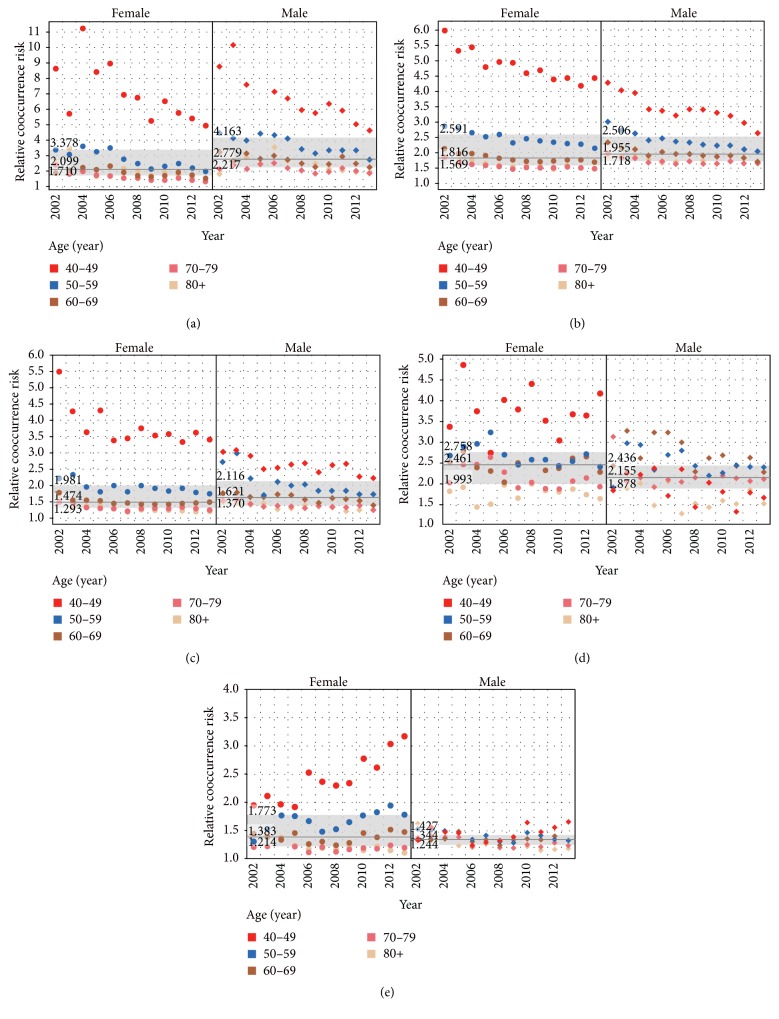
Trends in relative cooccurrence risks (RCoRs) for five major comorbidities over time by sex and age. Solid lines and gray bands show the median and interquartile ranges for RCoR for male and female patients, respectively. Numbers above or inside the band are upper quartile, median, and lower quartile of RCoR, respectively. (a) Disorders of lipid metabolism, (b) essential hypertension, (c) coronary atherosclerosis and other heart diseases, (d) chronic renal failure, and (e) acute cerebrovascular disease.

Because of the very large deviation in RCoRs for most comorbidities for patients aged under 40 years, trends in RCoRs over time were only analyzed for patients aged over 40 years.  Figure 4 shows trends in RCoR for diseases that could be considered as major comorbidities (both ACoR > 1% and RCoR > 1 statistically) for both male and female patients in any age group. The overall declining trends in relative risk rates were found for most major comorbidities associated with T2DM over time. In China, great efforts were made by government and communities in reducing the intake of salt, saturated fatty acids, and cholesterol, limiting cigarette advertising and ceasing smoking in public areas, promoting people's participation in sports, and controlling glucose levels, blood pressure, and lipid levels in adults with T2DM. All these may contribute to the reductions in the cooccurrence rates of EH, DLM, CHD, and CRF to some extent. Due to the larger rate of reductions among adults with diabetes than among adults without diabetes, the relative risks of these comorbidities associated with T2DM reduced. The results were similar with what were found in American adults with diabetes [39]. It was shown that acute myocardial infarction, stroke, end-stage renal disease, and lower-extremity amputation were reduced over time (from 1990 to 2010).

Male patients had a higher RCoR (median: 2.779; interquartile range: 2.217–4.163) of DLM than female patients (RCoR median: 2.099; interquartile range: 1.710–3.378, *P* = 0.001), while female patients had a higher RCoR (median: 2.461; interquartile range: 1.993–2.758) of CRF than male patients (RCoR median: 2.155; interquartile: 1.875–2.436; *P* = 0.009). For CHD, EH, and ACVD, no RCoR differences between male and female patients were found (*P* = 0.508, 0.235, and 0.255, resp.). DLM, EH, CHD, and ACVD showed declining associations with T2DM for patients aged <50, 50–59, and ≥60 years, respectively (*P* < 0.001, except for *P* < 0.05 for DLM in patients aged 50–59 and ≥60 years), while CRF showed a stronger association with T2DM for patients aged ≥60 years than that for patients aged <50 and 50–59 years, respectively (*P* < 0.001).

Dyslipidemia is a major risk factor for cardiovascular disease in diabetes, while in turn cardiovascular disease is the major cause of morbidity and mortality for individuals with diabetes. In this study, DLM had a relatively large influence on the population with T2DM (overall ACoR 12.5%) and showed a strong association with T2DM for both male and female patients and patients of any age (overall RCoR 3.477). Specifically, even though RCoRs declined over time for almost all major comorbidities for individuals of any age and sex, DLM remained the first strongest T2DM-associated comorbidity in men and in 40–49 years' age group over the entire study period. Therefore, DLM can be considered as the most severe comorbidity among men as well as middle-aged people with T2DM, suggesting that the control of dyslipidemia in middle-aged men is particularly important. In our study, differences in major macrocardiovascular risk factors (EH, CHD, and ACVD) in individuals with T2DM were slightly greater in men than women over time, which differs from the findings of previous studies [40–42]. EH and CHD, as two main chronic cardiovascular diseases, had similar changing trends in RCoR over time by sex and age. That is, men had higher risks than women, younger patients had higher risks than older patients, and risks declined from 2002 to 2013 for patients aged <60 years. However, ACVD showed different trends by sex, by age, and over time. Even women aged <60 years had a clearly increased risk of ACVD, a finding that warrants attention. In contrast to other comorbidities, chronic kidney failure showed distinctive trends. First, female patients had an obvious higher risk of CRF than men (median RCoR 2.461 versus 2.155, *P* = 0.009), which is consistent with other studies conducted in United States [43, 44]. Second, male patients aged 40–49 years had a lower risk of CRF than those aged 50–70. Finally, there was no increase or decrease in the risk of CRF in men or women over the study period. Patients with diabetes and kidney disease represent a special risk group as they have higher mortality than individuals with diabetes and normal kidney function. Therefore, these results highlight an urgent need for regular nephropathy screening among women with diabetes and diabetes patients aged >50 years, to help prevent progression to chronic renal disease.

### 3.4. T2DM as the Comorbid Disorder of Three Major Chronic Disorders

**Figure 5:**
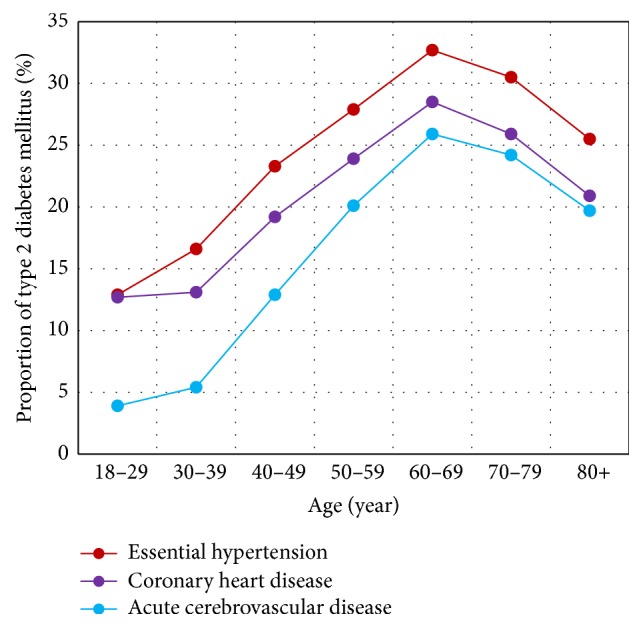
Proportion of patients having type 2 diabetes mellitus among hospitalized patients with essential hypertension, coronary heart disease, or acute cerebrovascular disease at different age groups.

It was interesting that T2DM was also among the most common comorbidities of EH, CHD, and ACVD, the diseases that were the most popular comorbidities of T2DM. T2DM were the first, second, and third popular comorbidities of EH, ACVD, and CHD (ACoR 29.8%, 23.0%, and 25.9%, resp.). Female patients with EH, CHD, or ACVD showed consistently higher proportions of having T2DM than male patients (32.4% versus 27.0%, 28.8% versus 22.8%, and 26.8% versus 20.1% with all *P* values <0.0001, resp.). Patients with EH, CHD, or ACVD also showed consistent trends in proportions of having T2DM at different age groups (Figure 5), where patients aged 60–69 years had the largest proportions (32.7%, 28.5%, and 25.9% for EH, CHD, and ACVD, resp.) of having a comorbidity of T2DM. The comorbid relationship among the four major chronic disorders had become a comprehensive and interactive web.

## 4. Conclusions

This EMR-based study has unique strengths compared with studies or surveys on smaller samples. First of all, available diagnoses are all based on actual administrative data collected as a part of usual clinical practice in the real-world setting, without any specific research purpose, resulting in a more cost-efficient study means. Second, diagnosis data are collected from all hospitals distributed throughout the city, providing data from a diverse population comprising rural and urban dwellers, the young and old, and men and women. Therefore, the background demographic characteristics of the study population are unlikely to be a source of bias. Last, large EMR database-based hospital discharge data used in this study captured a broad range of cooccurring T2DM and other disorders, providing an opportunity to comprehensively examine and characterize wide-ranging patterns of comorbidities in the real-world setting. Our work demonstrates how clinically derived data can be used to identify and track trends in T2DM prevalence and related comorbidities, and the findings may be important for administrators, clinicians, and researchers involved in the management of T2DM. This method may be widely applied to exploring other chronic disease-related comorbidities using EMR warehouses.

## Supplementary Material

Table S1. Absolute and relative co-occurrence risk for 27 major comorbidities of type 2 diabetes mellitus. Table S2. Absolute and relative co-occurrence risk of comorbidities of type 2 diabetes mellitus for men and women. Table S3. Absolute and relative co-occurrence risk of comorbidities of type 2 diabetes mellitus for all patients and patients aged 18-29 and 30-39.

## Figures and Tables

**Table 1:** 

Disease *X*	Disease *Y*		Total
	Present	Absent	
Present	*a*	*b*	*a* + *b*
Absent	*c*	*d*	*c* + *d*

Total	*a* + *c*	*b* + *d*	*n* = (*a* + *b* + *c* + *d*)
