# Supplementary material for: Comorbidity in Adult Patients Hospitalized with Type 2 Diabetes in Northeast China: An Analysis of Hospital Discharge Data from 2002 to 2013
Source: Biomed Res Int. 2016 Oct 25;2016:1671965. doi: 10.1155/2016/1671965 (PMC5099493; doi:10.1155/2016/1671965)
Supplement: Supplementary file 1 — Table S1. Absolute and relative co-occurrence risk for 27 major comorbidities of type 2 diabetes mellitus. Table S2. Absolute and relative co-occurrence risk of comorbidities of type 2 diabetes mellitus for men and women. Table S3. Absolute and relative co-occurrence risk of comorbidities of type 2 diabetes mellitus for all patients and patients aged 18-29 and 30-39. [file 1671965.f1.pdf]

Table S1. Absolute and relative co-occurrence risk for 27 major comorbidities of type 2 diabetes mellitus

Disease	CCS code	ACoR	RCoR
Thyroid disorders	48	2.8%	1.14
Nutritional deficiencies	53	12.5%	3.48
Fluid & electrolyte disorders	55	2.7%	1.60
Other nutritional, endocrine & metabolic disorders	58	1.7%	2.15
Cataract	86	3.0%	1.62
Other nervous system disorders	95	2.5%	1.62
Essential hypertension	98	58.4%	2.90
Acute myocardial infarction	100	2.1%	2.22
Coronary atherosclerosis & other heart disease	101	23.9%	2.39
Conduction disorders	105	1.1%	1.45
Cardiac dysrhythmias	106	6.9%	1.23
Congestive heart failure, nonhypertensivw	108	9.9%	1.77
Acute cerebrovascular disease	109	16.9%	2.04
Occlusion or stenosis of precerebral arteries	110	2.3%	3.41
Other & ill-defined cerebrovascular disease	111	6.5%	2.37
Transient cerebral ischemia	112	7.3%	1.75
Peripheral & visceral atheroscleros	114	3.3%	4.21
Pneumonia	122	4.5%	1.30
Acute bronchitis	125	2.3%	1.73
Other upper respiratory infections	126	2.6%	1.43
Other lower respiratory disease	133	4.9%	1.41
Biliary tract disease	149	4.5%	1.02
Other liver diseases	151	8.0%	1.99
Noninfectious gastroenteritis	154	1.3%	1.12
Chronic renal failure	158	4.4%	2.77
Urinary tract infections	159	6.5%	2.91
Skin & subcutaneous tissue infections	197	1.2%	1.88

CCS: Clinical Classifications Software; ACoR: absolute co-occurrence risk; RCoR: relative co-occurrence risk

Table S2. Absolute and relative co-occurrence risk of comorbidities of type 2 diabetes mellitus for men and women and overall

		Overall			Men			Women		
**Disease**	**CCS code**	**ACoR**	**RCoR**	**MC**	**ACoR**	**RCoR**	**MC**	**ACoR**	**RCoR**	**MC**
Thyroid disorders	48	2.76%	1.14	Y	1.79%	1.50	Y	3.60%	1.03	N
Other endocrine disorders	51	1.02%[#]	3.85	N	0.88%	3.57	N	1.15%	4.07	Y
Nutritional deficiencies	53	12.51%	3.48	Y	11.95%	3.79	Y	13.00%	3.26	Y
Fluid & electrolyte disorders	55	2.66%	1.60	Y	2.44%	1.41	Y	2.85%	1.79	Y
Other nutritional, endocrine & metabolic disorders	58	1.69%	2.15	Y	2.06%	1.98	Y	1.37%	2.45	Y
Deficiency and other anemia	59	1.72%	0.79	N	1.57%	1.16	Y	1.85%	0.64	N
Cataract	86	2.97%	1.62	Y	2.55%	1.37	Y	3.34%	1.85	Y
Other eye disorders	91	1.03%*	1.20	N	0.99%	1.02[&]	N	1.07%	1.39	Y
Other nervous system disorders	95	2.48%	1.62	Y	2.49%	1.37	Y	2.47%	1.94	Y
Heart valve disorders	96	0.91%	0.94	N	0.73%	0.80	N	1.07%	1.06	Y
Essential hypertension	98	58.39%	2.90	Y	53.61%	2.55	Y	62.57%	3.24	Y
Acute myocardial infarction	100	2.13%	2.22	Y	2.45%	1.77	Y	1.85%	3.20	Y
Coronary atherosclerosis & other heart disease	101	23.89%	2.39	Y	21.41%	2.03	Y	26.07%	2.73	Y
Conduction disorders	105	1.12%	1.45	Y	1.35%	1.38	Y	0.92%	1.57	N
Cardiac dysrhythmias	106	6.87%	1.23	Y	7.02%	1.12	Y	6.74%	1.36	Y
Congestive heart failure, Nnhypertensivw	108	9.90%	1.77	Y	8.60%	1.46	Y	11.05%	2.08	Y
Acute cerebrovascular disease	109	16.93%	2.04	Y	17.77%	1.73	Y	16.20%	2.48	Y
Occlusion or steNsis of precerebral arteries	110	2.34%	3.41	Y	2.79%	3.18	Y	1.95%	3.77	Y
Other & ill-defined cerebrovascular disease	111	6.49%	2.37	Y	7.34%	2.09	Y	5.74%	2.79	Y
Transient cerebral ischemia	112	7.31%	1.75	Y	7.06%	1.72	Y	7.52%	1.77	Y
Peripheral & visceral atheroscleros	114	3.25%	4.21	Y	4.04%	3.96	Y	2.56%	4.63	Y
Pneumonia	122	4.53%	1.30	Y	5.07%	1.20	Y	4.05%	1.43	Y
Acute bronchitis	125	2.25%	1.73	Y	2.15%	1.68	Y	2.34%	1.77	Y
Other upper respiratory infections	126	2.55%	1.43	Y	2.46%	1.31	Y	2.63%	1.54	Y
Chronic obstructive pulmonary disease and bronchiectasis	127	4.65%	0.93	N	5.09%	0.84	N	4.26%	1.06	Y
Other lower respiratory disease	133	4.94%	1.41	Y	5.56%	1.25	Y	4.39%	1.67	Y
Biliary tract disease	149	4.53%	1.02	Y	4.55%	1.00	N	4.51%	1.05	Y
Other liver diseases	151	7.97%	1.99	Y	9.17%	1.89	Y	6.92%	2.13	Y
Noninfectious gastroenteritis	154	1.33%	1.12	Y	1.24%	1.00	N	1.42%	1.23	Y

Chronic renal failure	158	4.42%	2.77	Y	4.61%	2.41	Y	4.25%	3.23	Y
Urinary tract infections	159	6.51%	2.91	Y	3.00%	1.85	Y	9.58%	3.44	Y
Hyperplasia of prostate	164	1.75%	1.20	N	3.75%	1.21	Y	/	/	N
Skin & subcutaneous tissue infections	197	1.21%	1.88	Y	1.59%	1.78	Y	0.87%	2.08	N

CCS: Clinical Classifications Software; ACoR: absolute co-occurrence risk; RCoR: relative co-occurrence risk; MC: Major comorbidity

# p-value = 0.132 compared to 1%; * p-value = 0.024 compared to 1%; & p-value = 0.861 compared to 1

Table S3. Absolute and relative co-occurrence risk of comorbidities of type 2 diabetes mellitus for all patients and patients aged 18-29 and 30-39

		Overall			18-29 years			30-39 years		
Disease	CCS code	ACoR	RCoR	MC	ACoR	RCoR	MC	ACoR	RCoR	MC
Thyroid disorders	48	2.76%	1.14	Y	3.02%	3.94	Y	2.95%	1.61	Y
Other endocrine disorders	51	1.02%#	3.85	N	1.88%	12.85	N	1.01%	4.50	Y
Nutritional deficiencies	53	12.51%	3.48	Y	24.57%	104.61	Y	24.27%	23.89	Y
Fluid & electrolyte disorders	55	2.66%	1.60	Y	3.57%	7.80	Y	2.37%	3.98	Y
Other nutritional, endocrine & metabolic disorders	58	1.69%	2.15	Y	7.98%	31.24	Y	4.66%	12.29	Y
Deficiency and other anemia	59	1.72%	0.79	N			N			N
Cataract	86	2.97%	1.62	Y	1.01%	7.31	Y	0.89%	3.50	N
Other eye disorders	91	1.03%*	1.20	N	1.25%	3.09	N	0.89%	2.19	N
Other nervous system disorders	95	2.48%	1.62	Y	0.96%	1.58	N	2.21%	2.26	Y
Heart valve disorders	96	0.91%	0.94	N			N			N
Essential hypertension	98	58.39%	2.90	Y	14.83%	33.93	Y	24.42%	10.03	Y
Acute myocardial infarction	100	2.13%	2.22	Y	0.61%	30.04	N	0.85%	6.24	N
Coronary atherosclerosis & other heart disease	101	23.89%	2.39	Y	2.04%	33.45	Y	3.20%	7.58	Y
Conduction disorders	105	1.12%	1.45	Y	0.73%	7.30	N	0.37%	2.47	N
Cardiac dysrhythmias	106	6.87%	1.23	Y	0.87%	2.40	N	1.32%	1.63	Y
Congestive heart failure, Nnhypertensivw	108	9.90%	1.77	Y	1.24%	7.34	Y	1.79%	3.65	Y
Acute cerebrovascular disease	109	16.93%	2.04	Y	2.12%	9.38	Y	2.42%	2.86	Y
Occlusion or steNsis of precerebral arteries	110	2.34%	3.41	Y	0.59%	74.49	N	0.81%	17.48	N
Other & ill-defined cerebrovascular disease	111	6.49%	2.37	Y	1.01%	15.71	Y	1.41%	5.78	Y
Transient cerebral ischemia	112	7.31%	1.75	Y	0.69%	11.89	N	1.85%	3.71	Y
Peripheral & visceral atheroscleros	114	3.25%	4.21	Y	0.56%	25.59	N	0.92%	14.90	N
Pneumonia	122	4.53%	1.30	Y	2.45%	1.69	Y	2.48%	1.45	Y
Acute bronchitis	125	2.25%	1.73	Y	1.17%	3.20	Y	1.59%	2.56	Y
Other upper respiratory infections	126	2.55%	1.43	Y	6.33%	5.10	Y	4.33%	2.83	Y
Chronic obstructive pulmonary disease and bronchiectasis	127	4.65%	0.93	N			N			N
Other lower respiratory disease	133	4.94%	1.41	Y	2.79%	2.19	Y	3.02%	2.60	Y
Biliary tract disease	149	4.53%	1.02	Y	5.19%	5.03	Y	5.83%	2.33	Y
Other liver diseases	151	7.97%	1.99	Y	22.00%	34.00	Y	19.49%	10.51	Y
Nninfectious gastroenteritis	154	1.33%	1.12	Y	1.87%	3.67	Y	1.12%	1.68	Y
Chronic renal failure	158	4.42%	2.77	Y	3.37%	5.14	Y	3.45%	3.25	Y
Urinary tract infections	159	6.51%	2.91	Y	6.09%	10.70	Y	4.41%	5.06	Y
Hyperplasia of prostate	164	1.75%	1.20	N	0.39%	59.57	N	0.30%	7.94	N
Skin & subcutaneous tissue infections	197	1.21%	1.88	Y	2.20%	6.30	Y	1.23%	3.04	Y

CCS: Clinical Classifications Software; ACoR: absolute co-occurrence risk; RCoR: relative co-occurrence risk; MC: Major comorbidity

# p-value = 0.132 compared to 1%; * p-value = 0.024 compared to 1%
